# Endoparasite survey in Amazonian manatees (*Trichechus inunguis*) under rehabilitation in the Peruvian Amazon

**DOI:** 10.1016/j.ijppaw.2024.101011

**Published:** 2024-10-28

**Authors:** Philipp Sziderics, Alessandra R. Medrano Zavala, Edmundo Guadalupe Parada Lopez, E. Leonardo Davila Panduro, Juan J.E. Sanchez Babilonia, Maria S. Unterköfler, David Ebmer, Emmanouil A. Koumantakis, Jim W. Ruiz Pezo, Franco I. Macedo Tafur, Luis A. Gomez-Puerta, Hans-Peter Fuehrer

**Affiliations:** a Institute of Parasitology, Department of Biological Sciences and Pathobiology, University of Veterinary Medicine Vienna, Austria; b Faculty of Human Medicine, Department of Medical Technology, National University of San Marcos, Lima, Peru; c Rainforest Awareness Rescue Centre - RAREC, Iquitos, Peru; d Centro de Rescate Amazónico – CREA, Iquitos, Peru; e Vienna Zoo, Vienna, Austria; f Universidad Nacional de la Amazonía Peruana, Iquitos, Peru; g Department of Veterinary Epidemiology and Economics, Universidad Nacional Mayor de San Marcos, Lima, Peru

**Keywords:** Amazonian manatee, Coccidiosis, Eimeria, *Eimeria trichechi*, Parasitology, Aquatic mammals, *Trichechus inunguis*

## Abstract

Manatee populations are declining worldwide, and all currently existing species are considered vulnerable by the IUCN. The most common problems during nurturing young Amazonian manatees, *Trichechus inunguis,* in rescue centres are of gastrointestinal nature leading to inappetence, diarrhoea, cachexia and even death. Endoparasites play an important role in the well-being of wildlife in captivity as well as in the wild, though information about relevant protozoan and metazoan endoparasites in Amazonian manatees is still scarce. Therefore, this study aimed to find endoparasites in *T. inunguis* by analyzing faecal samples from 23 Amazonian manatees which were kept in rescue centres in the Peruvian Amazon. The samples were screened for protozoan and metazoan parasites using coproscopical analysis and molecular tools. Out of twenty juvenile animals eleven were positive for at least one Eimeriidae. Two morphologically different, not yet genetically described *Eimeria* species were identified. One of them seems to be *Eimeria trichechi* which has only been described once in 1984 in Amazonian manatees from Brazil. It was not found to lead to clinical symptoms of coccidiosis in this study. The second, *Eimeria* sp. Type B was associated with clinical coccidiosis in a young Amazonian manatee, which showed gastrointestinal symptoms including diarrhoea, inappetence and cachexia. No other protozoan or metazoan parasite were detected in any of the samples. The present study is the first to investigate endoparasites in Amazonian manatees using molecular tools and is the first to identify an Eimeria species that could be associated with clinical symptoms in *T. inunguis*. With information from our study rescue centres can improve monitoring of parasites more effectively to reduce morbidity and mortality rates among rehabilitated manatees as well as improve the health status and fitness of animals for a successful release back into the wild.

## Highlights

• Information about relevant protozoan and metazoan endoparasites in Amazonian manatees is scarce.
• Two morphologically different, not yet genetically described *Eimeria* species were identified.
• *Eimeria* sp. Type B was associated with clinical coccidiosis in a young Amazonian manatee.

## Graphical abstract

**Figure:**
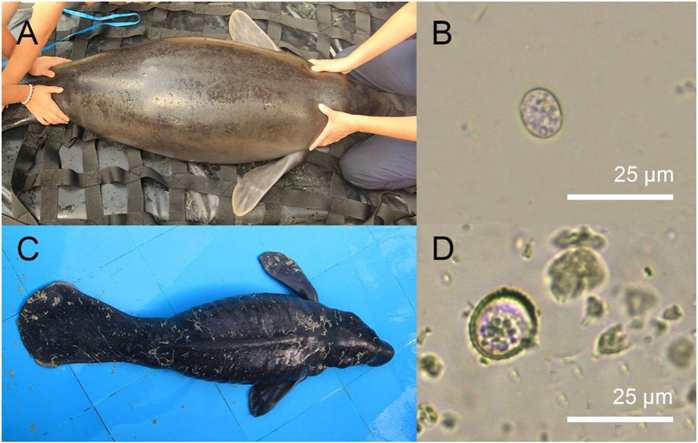

## 1 Introduction

Within the vastly neglected field of marine mammal parasitology the endoparasite fauna of manatees, especially the Amazonian and West African manatee has barely been studied. The number of mature Amazonian manatees has been decreasing over the last decades and the IUCN lists them as vulnerable (Marmontel et al., 2016). Due their cryptic behaviour and the complexity their habitat, the river systems of the Amazon rainforest, the exact number of Amazonian manatees, *Trichechus inunguis,* is still unknown, emphasizing the need for science-based conservation efforts. *T*. *inunguis* can be found throughout the whole Amazon Basin with its habitat including the Amazon River and its tributaries (Bonvicino et al., 2020). Amazonian manatees, like all the other species of the family of the Trichechidae are fully aquatic monogastric non ruminant herbivores (Calvimontes and Marmontel, 2022). There are many factors which are responsible for the loss of manatees worldwide with anthropogenic effects such as pollution (Silva Junior et al., 2022), loss of habitat, dam constructions (Arraut et al., 2017) and hunting having the greatest impact (Antunes et al., 2016).

Parasitology plays a crucial role in the conservation of many terrestrial and aquatic mammals (Zatrak et al., 2022). Juvenile manatees, being more vulnerable due to their developing immune systems, are particularly susceptible to parasitic infestations which can lead to malnutrition, anaemia, and secondary infections. Not many endoparasites of Amazonian manatees are known today emphasizing the need for more research in this field to enhance conservation efforts. Until now infections with the protozoan parasites *Giardia* spp. and *Cryptosporidium* spp. are known from Amazonian manatees in Brazil which was reported to lead to diarrhoea in Greater Caribbean manatees. Although clinical symptoms have not yet been described in *T. inunguis* (Borges et al., 2011). It is hypothesized that these parasites were most likely introduced into the habitat of the studied animals by sewage and wastewater from human civilization, adding another potential anthropogenic effect threatening wild manatee populations (Borges et al., 2016). Three different species of the genus *Eimeria* have been found in Greater Caribbean, Florida, and Amazonian manatees so far and these had the highest prevalence of parasitic infections in both wild and captive populations (Wyrosdick et al., 2018; Velez et al., 2019). The genus *Eimeria* is represented with over 1800 different species and a vast variety of subspecies. It has been found in a broad spectrum of mammalian and non-mammalian hosts, but in contrast to *Giardia* spp. and *Cryptosporidium* spp., they are very host specific (Duszynski and Upton, 2001). Infections have been reported to lead to clinical symptoms due to gastrointestinal dysfunction leading to dehydration, diarrhoea and inappetence in many species (Yun et al., 2000). In general, *Eimeria* infections are more frequent in younger herbivorous animals compared to older ones, since homologous reinfections usually lead to immunological protection in adult animals (Hermosilla et al., 2012). All *Eimeria* species replicate in the intestines of their hosts and shed their oocysts via faeces (Kemp et al., 2013). The coccidians of the Eimeriidae family are monoxenous and due to their high reproduction rates cause significant damage to the intestines of their hosts, leading to acute enteritis (Yun et al., 2000). *Eimeria* species that were found in manatees include *Eimeria manatus* and *Eimeria nodulosa* in faecal samples from the Greater Caribbean manatee, *Trichechus manatus manatus* and the Florida manatee, *Trichechus manatus latirostris* (Wyrosdick et al., 2018) and *Eimeria trichechi*, which has only been documented in *T. inunguis* from Brazil (Lainson et al., 1983). In Amazonian manatees from Peru three different Eimeriidae were characterized morphologically (Ruiz-Pezo et al., 2024).

Metazoan parasites have not been detected in Amazonian manatees yet, but many Trematode and Nematode species were found in Greater Caribbean and Florida manatees (Beck and Forrester, 1988; Mignucci-Giannoni et al., 1999; Colon-Llavina et al., 2009; Bando et al., 2013). The parasite fauna of Greater Caribbean manatees, especially ones found in Brazil are of great importance, since in this region the habitats of Amazonian and Greater Caribbean manatees overlap, and hybridization between the two species here indicates an exchange not only of genes, but possibly also of pathogens (Oliveira Luna et al., 2021).

The digenea *Pulmonicola cochleotrema* of the family Opisthotrematidae has been shown to have a high prevalence of 25–30% around the northern part of the Caribbean, especially in populations inhabiting the coastal waters around Florida and Puerto Rico (Beck and Forrester, 1988; Mignucci-Giannoni et al., 1999). A correlation of water salinity and the presence of *P. cochleotrema* is thought to exist. Unfortunately, coproscopical analysis has not proven to be a reliable test to screen for these parasites (Carvalho et al., 2009). In most cases, no symptoms are described, but infections have been reported to cause rhinitis, pneumonia, pulmonary oedema and can even lead to death (Beck and Forrester, 1988). The exact intermediate host is still unknown, but it is hypothesized that infected molluscs or crustaceans living on sea grass are ingested and are therefore able to infect manatees (Borges et al., 2016).

Another trematode that has been reported in Greater Caribbean manatees in the rivers Carare and San Juan in the Colombian Andean region is *Nudacotyle undicola* from the superfamily of the Pronocephaloidae. They colonize the small intestine, but no symptoms associated with this parasite have been reported so far (Beck and Forrester, 1988). Intermediate hosts are also still unknown for this trematode species.

Other trematode species from the family Paramphistomoidae that were reported from manatees are *Chiorchis fabaceus* and *C. groschafti*. They have been documented in both, *T. m. manatus* and *T. m. latirostris* (Bando et al., 2013). Intermediate hosts and life cycles of these parasites are unfortunately still unknown and further studies will be needed to better understand their parasite – host relations.

*Moniligirum blairi* from the family Opisthotrematidae has been examined in Greater Caribbean and Florida manatees around Florida and Mexico, but not further to the south (Dailey et al., 1988).

The only nematode that has been described to parasitize manatees is *Heterocheilus tunicatus* from the superfamily Ascaridoidea. It has been identified in *T. m. manatus* and *T. m. latirostris* in the Dominican Republic and Puerto Rico. No clinical symptoms have so far been associated with this parasite (Mignucci-Giannoni et al., 1999; Colon-Llavina et al., 2009).

Since the parasitic fauna of manatees depends not only on the manatee species or subspecies, but also on the location of the animals, it is of great importance to investigate manatees in various regions in order to create a better overview of the parasitic diversity in different populations. Such data will greatly improve the understanding of threats to these vulnerable animals.

Most of the rescued animals are calves usually found motherless, injured, or are confiscated by authorities after being kept as pets illegally (Calvimontes and Marmontel, 2022). The most common disease of calves at rescue centres is associated with the gastrointestinal tract, usually showing symptoms such as diarrhoea, tenesmus, restless swimming, constipation and inappetence (Bossart et al., 2003; Lazzarini et al., 2014). This emphasizes the need for more research to be done on enteropathogens including endoparasites of Amazonian manatees and enteropathogens in general to support conservation efforts (Neto et al., 2016).

This study is the first survey of endoparasites of Amazonian manatees in Peru using molecular tools. We aimed to create more data to support conservation measures for *T. inunguis* and to better understand the potential threats for this ecologically highly important species (Chavez et al., 2023) of the Amazon region. With more knowledge on relevant endoparasites and their life cycles, rescue centres can monitor and manage parasite loads more effectively to reduce morbidity and mortality rates among rehabilitated manatees as well as improve the health status and fitness of animals for a successful release back into the wild, since endoparasites play a significant role in the survival rates of wild animals treated in rescue centres (Zatrak et al., 2022). Moreover, understanding the parasitic fauna in these aquatic environments aids in the development of targeted treatment protocols and contributes to the broader ecological knowledge necessary for the long-term conservation of populations of Amazonian manatees.

## 2 Materials and methods

**Fig. 1:**
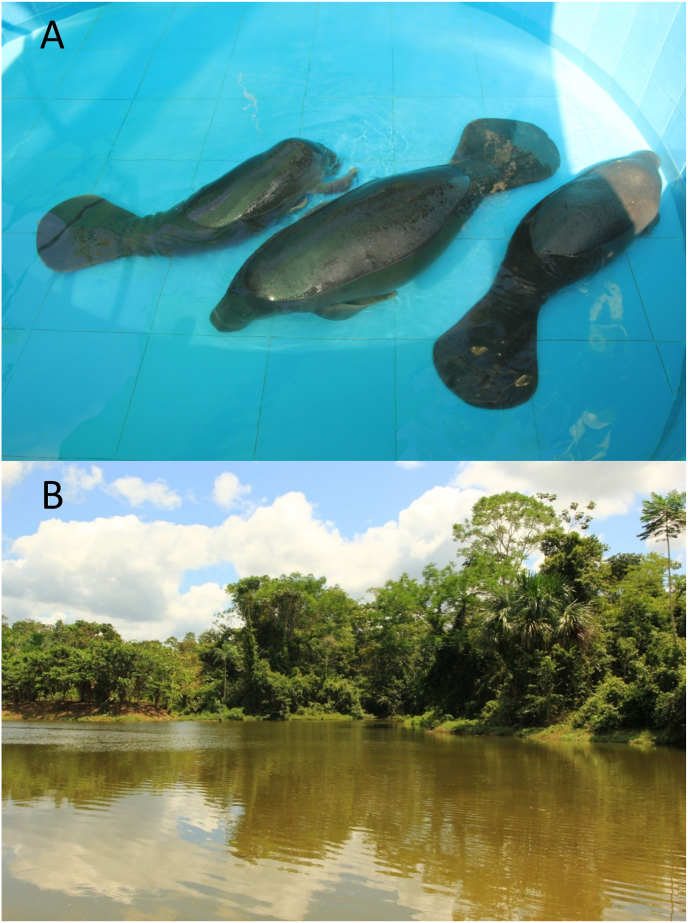
(A) Pond where adult manatees were kept, which were sampled in the present study. (B) Pools in which the juvenile manatees were kept in.

Faecal and blood samples were collected from 23 Amazonian manatees living in the Rescue Centres CREA (Centro de Rescate Amazónico) and RAREC (Rainforest Awareness Rescue Centre) in 2021 and 2023 in the surrounding area of Iquitos in the Peruvian department of Loreto. All the animals were found injured, motherless or were confiscated in the area around Iquitos and were kept in pools and ponds until a release back into the wild was possible. Of the 23 manatees 20 were under 1,5 years old and still receiving milk multiple times a day. These lived in pools built close to the forest either alone or in small groups of three, where the water was changed weekly, but was not filtered in any way (Fig. 1A). The three adult manatees were kept together in a big pond with possible contact to wildlife (Fig. 1 B).

One of the juvenile *T*. *inunguis* showed symptoms of gastrointestinal disease including diarrhoea, low body condition score and inappetence. The other 22 manatees were clinically healthy and did not show any gastrointestinal symptoms. None of the animals received antiparasitic treatment before samples were taken. All samples were collected during routine check-ups, for which they were taken out of the pool or pond with nets. Soon after the manatees were on land most of them started defecating. In animals that did not defecate spontaneously, gentle pressure was applied on their abdomen to not unnecessarily prolong surface times. Samples were stored in 125 ml plastic containers and kept cool at 4 °C until further diagnostics. Since previous studies had shown that coproscopical examination was not a reliable diagnostic test to detect infections with *Pulmonicola cochleotrema*, and opening the nasal cavities was not an option in the present study, the nostrils were inspected while the animals took a breath. Animals were screened for typical symptoms of *P. cochleotrema* infections including rhinitis and abnormal breathing sounds (Beck and Forrester, 1988; Borges et al., 2011). Blood samples were taken from the *Vena ulnaris* and stored in serum tubes until further processing to detect possible haemoparasites. Blood smears were performed and one smear from each animal was left unstained whereas a second one was stained with Diff-Quik before being examined under the microscope.

For the diagnostics of the faecal samples coproantigen-ELISAs to detect *Giardia* spp. (FASTest® Giardia Strip, Megacor, Hörbranz, Austria) and *Cryptosporidium* spp. (FASTest® Crypto Strip, Megacor) were initially performed. Subsequently, the samples were subjected to the Sheather's sedimentation, flotation (SSF)-technique using Sheather's sugar solution and examined by light microscopy. The samples were photographed using a digital camera.

Finally, DNA was extracted from all the faecal samples (NucleoSpin® Soil kit, Macherey-Nagel, Düren, Germany), and was used for PCR analysis. And all the protocols listed in (Table 1) were performed for every sample. All samples underwent PCR testing for *Enterocytozoon* sp. with nested ITS rRNA protocol, *Giardia* spp. with nested β Giardin, *Cryptosporidium* spp. with nested 18S RNA, *Eimeria* spp. with nested 18S RNA, Nematodes with ITS protocol and Trematodes using COI Neod protocol. All protocols that were used are also described in detail in (Table 1, Newton et al., 1998; Xiao et al., 1999; Sulaiman et al., 2004; Tseng et al., 2014; Duscher et al., 2015; Yang et al., 2016).

**Table 1:** List of PCR primers that were used for detection of endoparasites.

Pathogen	Gene	PCR method	Primer name	Sequence (5′-3′)	Den (T°/time)	Ann (T°/time)	Ext (T°/time)	Cycles
***Enterocytozoon* sp.**	ITS rRNA	Nest 1, forward	AL4037	GAT GGT CAT AGG GAT GAA GAG CTT	94°/45s	55°/45s	72°/1min	36x
		Nest 1, reverse	AL4039	ACG GAT CCA AGT GAT CCT GTA TT				
		Nest 2, forward	AL4038	AGG GAT GAA GAG CTT CGG CTC TG	94°/45s	55°/45s	72°/1min	36x
		Nest 2, reverse	AL4040	AGT GAT CCT GTA TTA GGG ATA TT				
***Giardia* spp.**	β Giardin	Nest 1, forward	β Giardin G7	AAG CCC GAC GAC CTC ACC CGC AGT GC	94°/30s	65°/30s	72°/1min	36x
		Nest 1, reverse	β Giardin G759 R1	GAG GCC GCC CTG GAT CTT CGA GAC GAC				
		Nest 2, forward	β Giardin F2	GAA CGA ACG AGA TCG AGG TCC G	94°/30s	65°/30s	72°/1min	36x
		Nest 2, reverse	β Giardin R2	CTC GAC GAG CTT CGT GTT				

Pathogen	Gene	PCR method	Primer name	Sequence (5′-3′)	Den (T°/time)	Ann (T°/time)	Ext (T°/time)	Cycles
***Cryptosporidium* spp.**	18S RNA	Nest 1, forward	CrySSUF1	TTC TAG AGC TAA TAC ATG CG	95°/40s	60°/40s	72°/1.5min	36x
		Nest 1, reverse	CrySSUR1	CCC ATT TCC TTC GAA ACA GGA				
		Nest 2, forward	CrySSUF2	GGA AGG GTT GTA TTT ATT AGA TAA AG	95°/40s	55°/40s	72°/1.5min	36x
		Nest 2, reverse	CrySSUR2	CTC ATA AGG TGC TGA AGG AGT A				
***Eimeria* spp.**	18S RNA	Nest 1, forward	EiGTF1	TTC ACT GGT CCC TCC GAT C	94°/30s	58°/30s	72°/30s	36x
		Nest 1, reverse	EiGTR1	AAC CAT GGT AAT TCT ATG G				
		Nest 2, forward	EiGTF2	TTA CGC CTA CTA GGC ATT CC	94°/30s	58°/30s	72°/30s	36x
		Nest 2, reverse	EiTR2	TGA CCT ATC AGC TTT CGA CG				
**Nematoda**	ITS	forward	NC5	GTA GGT GAA CCT GCG GAA GGA TCA TT	94°/30s	55°/40s	72°/1min	36x
		reverse	NC2	TTA GTT TCT TTT CCT CCG C				
**Trematoda**	COI Neod	forward	COI_Neod_Fw	TTT ACT TTG GAT CAT AAG CG	95°/1min	48°/1min	72°/1min	35x
		reverse	COI_Neod_Rv	CAA AAA AAC CAA AAC ATA TGT TGA A				

After the PCRs, sequencing was performed on all positive samples using the Sanger method (Microsynth Seqlab, Germany). Sequences obtained in this study of *Eimeria* spp. were phylogenetically compared to other sequences available at the NCBI GenBank database of *Eimeria* spp. from manatees. A preliminary tree was calculated to identify a suitable clade for the calculation of the median-joining haplotype network. The tree was calculated based on 375 sequences (1127 nucleotide positions) using the W-IQ-TREE web server (http://iqtree.cibiv.univie.ac.at/, Trifinopoulos et al., 2016). All sequences of manatees were within one clade (see Supplements 1). The sequences were aligned and sorted using the default option (FFT-NS-2) in MAFFT v.7.311 (Katoh and Standley, 2013). Median-joining haplotype network was calculated with Network 10.2.0.0 (Fluxus Technology Ltd., Suffolk, UK), applying the default settings. The network was graphically prepared in Network Publisher v.2.1.2.5 (Fluxus Technology Ltd., Suffolk, UK) and finalized with CorelDRAW 2023 (Corel, Ottawa, Canada). Sequences used for analysis were uploaded to GenBank.

## 3 Results

Overall, 55% (11/20) of the nursing juvenile manatees included in the present study were found to be infected with at least one protozoan species, whereas no infections were seen in any of the adult manatees. None of the blood samples showed any evidence of the presence of haemoparasites. Neither coproantigen testing, nor coproscopical examination, nor PCR showed the presence of *Giardia* sp. or *Cryptosporidium* sp. in any of the samples. Coproscopical analysis and PCR testing for detection of nematodes and trematodes were negative in all samples as well, thus no metazoan parasites were found in any of the animals that were sampled in this study. However, during coproscopical analysis coccidia, more precisely members of the genus *Eimeria*, were found in eleven of the twenty juvenile animals sampled in the present study.

**Fig. 2:**
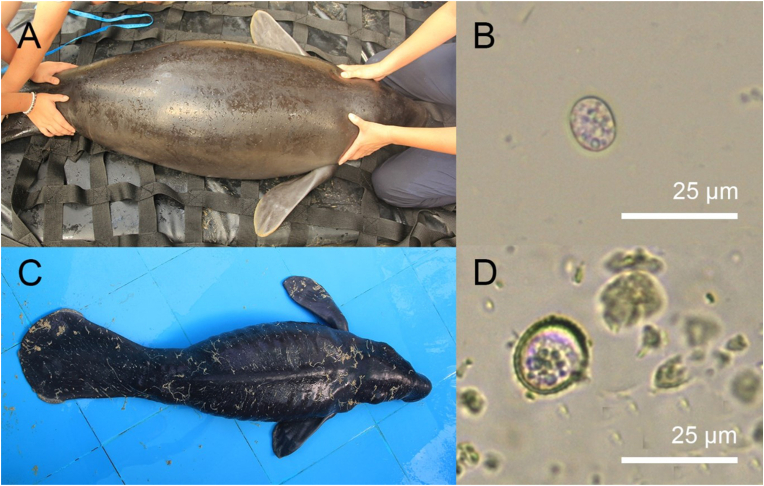
(A) Clinically healthy juvenile Amazonian manatee (*Trichechus inunguis*) infected with *Eimeria* cf. *trichechi.* (B) *Eimeria* cf. *trichechi* with thin, uni-layered oocyst wall. (C) Juvenile manatee with symptoms of coccidiosis, including diarrhoea, inappetence and cachexia. (D) *Eimeria* sp. Type B, an Eimeria with a thick multilayered oocyst wall.

Morphologically two different *Eimeria* sp. could be identified. *Eimeria* sp. Type A was present in eleven of the twenty juvenile animals and had a size of 12.59 × 11.24 μm (10.45-13.72 x 9.92–13.4). The oocyst wall was thin, uni-layered and four ellipsoidal sporocysts could be identified (Fig. 2B). These animals were all clinically healthy (Fig. 2A). The second *Eimeria*, *Eimeria* sp. Type B, was found in coproscopical analysis in samples from one juvenile animal. It had a mean size of 17.82 × 15.68 μm (16.25-18.9 x 14.48–17.03) and had a thicker, multilayered oocyst wall (Fig. 2D). *Eimeria* sp Type A and *Eimeria* sp. Type B were both found in samples from this manatee which displayed gastrointestinal symptoms including diarrhoea, cachexia and inappetence (Fig. 2C).

**Fig. 3:**
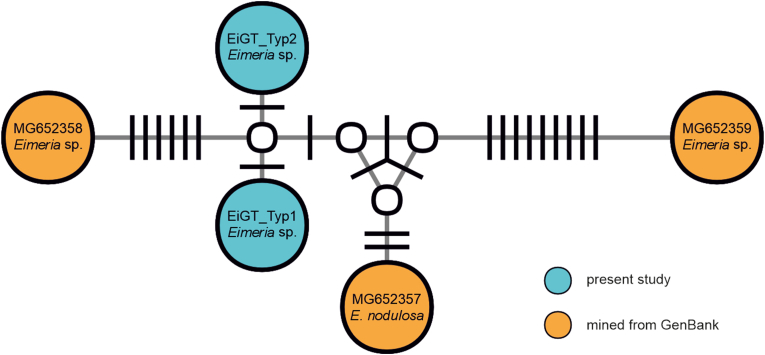
Median-joining haplotype network of the 18S ribosomal RNA sequences (1193 nucleotide positions) of *Eimeria* spp. reported from manatees (*Trichechus manatus*). Circles represent haplotypes. GenBank accession numbers and species of the haplotypes are shown in the circles; white circles represent intermediate nodes; bars on branches connecting haplotypes represent the number of substitutions.

DNA sequencing revealed two different haplotypes of *Eimeria* sp. named EiGT_Type 1 and EiGT_Type 2. EiGT_Type 1 was detected in 7 animals, EiGT_Type 2 was detected in 5 animals and in one animal the sequence was too short to determine the haplotype. The gene sequences of EiGT_Type 1 and EiGT_Type 2 were uploaded to GenBank database with the accession numbers PP475141 and PP475142. The results from the genetic sequencing were displayed in a phylogenetic network. Close relationships were detected between the Eimeria species from the present study and three Eimeria species that have been previously described in Greater Caribbean manatees from northern Colombia (Fig. 3).

## 4 Discussion

Knowledge regarding the parasite fauna of manatees throughout the Amazon Basin is scarce. To the best of our knowledge, the present study is the first to use molecular tools to investigate faecal samples of Peruvian *T. inunguis* for endoparasites and the first to analyse blood samples. We report eleven cases of *Eimeria* infections and provide two sequences of *Eimeria* sp. infecting *T. inunguis* as well as two morphologically different *Eimeria* sp. infecting juvenile Amazonian manatees.

In the present study, *Eimeria* cf. *trichechi* was found in 55% (11/20) of the juvenile *T. inunguis*. It presented morphological similarities to *Eimeria trichechi*, which has been reported in adult Amazonian manatees from Brazil (Lainson et al., 1983). To our knowledge, this *Eimeria* species has not been detected in a study since then. Ten of the eleven *E*. cf. *trichechi* infected *T. inunguis* did not show any clinical symptoms of coccidiosis. The one animal that did show gastrointestinal symptoms including inappetence, diarrhoea and cachexia, had a co-infection with a second *Eimeria* sp. Lainson et al. (1983) did not report any gastrointestinal symptoms in adult *T. inunguis* infected with *E. trichechi* either. The similar morphology, and the similar size make it very likely that the *Eimeria* species we found is *E. trichechi* or at least very closely related. Unfortunately, no genetical sequencing has been done for *E. trichechi* so far. Thus, we propose that the present study is the first to provide genetical data on this *Eimeria* species and is also the first to report *E*. cf. *trichechi* in Amazonian manatees in Peru. It would be of great interest to screen more *T. inunguis* in different regions across the Amazon Basin in future studies for *E*. cf. *trichechi* to better understand the distribution and possible influences these protozoans can have on juvenile and adult Amazonian manatees not only in captivity, but also in the wild.

The second *Eimeria* species was sampled in a juvenile *T. inunguis* which showed clinical symptoms of coccidiosis such as inappetence, diarrhoea and cachexia. We hypothesize that the infection with this *Eimeria* sp. Type B can be associated with these clinical symptoms since this animal was the only one that showed symptoms of coccidiosis and no other animal included in this study was positive for *Eimeria* sp. Type B. However, additional reports and studies are needed to prove that *Eimeria* sp. Type B can lead to clinical coccidiosis in juvenile Amazonian manatees. Furthermore, gastrointestinal problems are very frequent in rescued manatee calves due to stress and feed change as well as problems with digesting milk replacer, so diarrhoea and inappetence are very common (Calvimontes and Marmontel, 2022). Nevertheless, many studies have already shown that *Eimeria* sp. infections can lead to the previously described symptoms in many different terrestrial and aquatic mammals. This indicates that infections with *Eimeria* sp. Type B could lead to symptoms of clinical coccidiosis in juvenile Amazonian manatees.

Previous studies in Greater Caribbean manatees have shown high rates of coccidiosis in juvenile manatees opposed to a lower prevalence of *Eimeria* in adult manatees in Colombia as well (Velez et al., 2018, 2019). Thus, our data are in agreement with these observations: 55% (11/20) of juvenile, milk drinking, manatees tested positive for *Eimeria* sp., whereas none of the adult manatees was infected with any protozoan parasite. However, it is important to consider that the juvenile animals in our study were all kept in pools where constant reinfections of the manatees happen much more frequently than in the adult animals that were kept in a relatively big pond, lowering the chances for reinfections. In contrast, in another study on Amazonian manatees from Peru 10 out of 10 animals of all ages were infected with at least one *Eimeria* sp. (Ruiz-Pezo et al., 2024). Similarly, Lainson et al. (1983) found a 64% (9/14) prevalence of *E*. *trichechi* in adult Amazonian manatees in northern Brazil, but it is important to note that no juvenile animals were included in that study. Nevertheless, in general, *Eimeria* infections are more frequent in younger herbivorous animals compared to older ones, since homologous reinfections usually lead to immunological protection in adult animals in (Taubert et al., 2009; Hermosilla et al., 2012).

Additionally, by constructing a phylogenetic network with the genetical data, that we gathered we were able to show close relations to *Eimeria* species that have been found in Greater Caribbean manatees in the rivers Carare and San Juan in northwestern Colombia. This region is approximately 2500 km away from Iquitos, Peru where the samples from the present study were gathered and no direct contact of manatee populations from these regions is possible. For future studies more genetical data of *Eimeria* species along the northern coast of South America and throughout the Amazon Basin should be gathered. This could be added to the phylogenetic network, allowing *Eimeria* sp. relations in South American manatees to be better understood and conclusions about possible contact between different manatee populations to be drawn. The sequences obtained in the present study clustered between the sequences assigned to *Eimeria nodulosa* and an unassigned *Eimeria* sp. from another study (Velez et al., 2019). This phylogenetical close relationship makes it difficult to assign a species solely on the basis of 18S sequences. Other genetic markers might be better suited and morphological characterization is crucial for the determination of species. However, it must be considered, that co-infection might occur, even if not detected by coproscopical examination.

No other endoparasites were found in any of the samples which could be due to the rather small sample size of 23 animals. Similar to previous studies in Greater Caribbean and Florida manatees, none of the faecal samples from the 23 *T. inunguis* in this study were positive for *Enterocytozoon* sp. infections (Velez et al., 2018, 2019; Wyrosdick et al., 2018; Attademo et al., 2020). These samples were also negative for *Giardia* spp. and *Cryptosporidium* spp. which have both been detected in free ranging and captive Amazonian manatees in northern Brazil (Borges et al., 2017). Even though the frequent direct contact to humans, especially considering the juvenile animals which were fed by hand using bottles multiple times a day, would presumably lead to a higher probability of infection with such zoonotic endoparasites. A possible explanation for the absence of these zoonotic protozoans in the present study are the geographic circumstances of the areas. The population density in these areas is much lower than in northern Brazil (Tritsch et al., 2016) presumably leading to a lower infection pressure of zoonotic pathogens on aquatic animals given that pollution with sewage or domestic animal faeces can act as a pathway to infect wildlife with zoonotic parasites (Fayer et al., 2014). Of course, more studies must be conducted and much more data be gathered to support such a hypothesis. Metazoan parasites have not been found in any of the samples included in the present study either. It is known that in wild Greater Caribbean manatees the prevalence of trematode and nematode species are generally low (Velez et al., 2018). Furthermore, no possible intermediate hosts are known for any potential metazoan parasites infecting Amazonian manatees. Therefore, it is possible that none of the animals included in the present study had contact to potential intermediate hosts, especially considering the captive environment in which the juvenile animals were kept in. Even though the pond where the adult manatees were kept was very much like their natural habitat, surrounded by lush vegetation and dense with aquatic plants only three animals included in this study lived there which may be too small for a sample size. Especially considering the low prevalences of metazoan parasites in Greater Caribbean manatees (Beck and Forrester, 1988; Mignucci-Giannoni et al., 1999; Velez et al., 2019). Lastly none of the blood samples showed any evidence of haemoparasites in Amazonian manatees in northern Peru. This is not surprising since there are no known haemoparasites, neither from *T. manatus,* nor from *T. inunguis*. Furthermore, no hematophagous ectoparasites which would be needed as vectors are known in any manatee species.

In summary, with the present study we were able to report the first clinical case of coccidiosis in Amazonian manatees. Furthermore, we could provide sequence data from two Eimeriidae and were also able to describe two morphologically different *Eimeria* sp., one of which, to our knowledge, was not known before. Our study can be used as a baseline for future studies on manatee populations in the Amazon Basin. More research is needed especially in free ranging Amazonian manatees to better understand the parasite fauna and their impact on manatee health as well as the epidemiological aspects of parasitosis. It is of great importance for conservation efforts to acquire more information on pathogens of *T. inunguis* in wild and captive populations and more interdisciplinary work is needed in favour of the One Health concept to protect this vulnerable species.

## CRediT authorship contribution statement

**Philipp Sziderics:** Writing – review & editing, Writing – original draft, Validation, Methodology, Investigation, Formal analysis, Data curation, Conceptualization. **Alessandra R. Medrano Zavala:** Writing – review & editing, Methodology, Investigation. **Edmundo Guadalupe Parada Lopez:** Writing – review & editing, Methodology, Investigation, Data curation. **E. Leonardo Davila Panduro:** Methodology, Investigation, Data curation. **Juan J.E. Sanchez Babilonia:** Writing – review & editing, Methodology, Investigation, Data curation. **Maria S. Unterköfler:** Writing – review & editing, Visualization, Validation, Methodology, Data curation. **David Ebmer:** Writing – review & editing, Supervision, Methodology, Conceptualization. **Emmanouil A. Koumantakis:** Writing – review & editing, Methodology, Investigation, Data curation. **Jim W. Ruiz Pezo:** Writing – review & editing, Methodology, Investigation, Data curation. **Franco I. Macedo Tafur:** Writing – review & editing, Methodology, Investigation, Data curation. **Luis A. Gomez-Puerta:** Writing – review & editing, Supervision, Methodology, Data curation, Conceptualization. **Hans-Peter Fuehrer:** Writing – review & editing, Supervision, Project administration, Methodology, Investigation, Formal analysis, Conceptualization.

## Availability of data and materials

The data presented in this study are contained within the article and supplementary material (Supplementary Files). Additional data can be provided on request.

## Funding

This research received no external funding.

## Declarations of interest

None.
