# Supplementary figures and images for: Endoparasite survey in Amazonian manatees (Trichechus inunguis) under rehabilitation in the Peruvian Amazon
Source: Int J Parasitol Parasites Wildl. 2024 Oct 28;25:101011. doi: 10.1016/j.ijppaw.2024.101011 (PMC11550573; doi:10.1016/j.ijppaw.2024.101011)

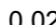

Supplement: Multimedia component 1 [file mmc1.pdf]
